# The Promise of Circulating Tumor DNA in Head and Neck Cancer

**DOI:** 10.3390/cancers14122968

**Published:** 2022-06-16

**Authors:** Sukhkaran S. Aulakh, Dustin A. Silverman, Kurtis Young, Steven K. Dennis, Andrew C. Birkeland

**Affiliations:** 1 School of Medicine, University of California, Davis, CA 95817, USA; ssaulakh@ucdavis.edu; 2 Department of Otolaryngology—Head and Neck Surgery, University of California, Davis, CA 95817, USA; dsilverman@ucdavis.edu (D.A.S.); skdennis@ucdavis.edu (S.K.D.); 3 John A. Burns School of Medicine, Honolulu, HI 96813, USA; kcyoung@hawaii.edu

**Keywords:** head and neck cancer, liquid biopsy, circulating tumor DNA, head and neck squamous cell carcinoma, exosomes, gene methylation

## Abstract

**Simple Summary:**

Head and neck cancer remains a challenging and deadly disease as it is often identified in more advanced stages due to limitations in screening and surveillance. Circulating tumor DNA (ctDNA) has the potential to improve outcomes by enhancing screening, early diagnosis, and surveillance in head and neck cancer patients. In this review, we discuss the current state of the literature using ctDNA as a biomarker for head and neck cancer screening, diagnosis, treatment response, and prognosis.

**Abstract:**

As the seventh most common cancer globally, head and neck cancers (HNC) exert considerable disease burden, with an estimated 277,597 deaths worldwide in 2020 alone. Traditional risk factors for HNC include tobacco, alcohol, and betel nut; more recently, human papillomavirus has emerged as a distinct driver of disease. Currently, limitations of cancer screening and surveillance methods often lead to identifying HNC in more advanced stages, with associated poor outcomes. Liquid biopsies, in particular circulating tumor DNA (ctDNA), offer the potential for enhancing screening, early diagnosis, and surveillance in HNC patients, with potential improvements in HNC patient outcomes. In this review, we examine current methodologies for detecting ctDNA and highlight current research illustrating viral and non-viral ctDNA biomarker utilities in HNC screening, diagnosis, treatment response, and prognosis. We also summarize current challenges and future directions for ctDNA testing in HNC patients.

## 1. Introduction

Globally, 562,328 people were diagnosed with head and neck cancer (HNC) in 2020 with an estimated 277,587 deaths due to the disease [1]. These cancers incorporate the mucosa of the upper aerodigestive tract, most commonly involving the nasopharynx, oropharynx, oral cavity, hypopharynx or larynx. HNC is predominantly driven by environmental exposures such as tobacco, alcohol, and viral exposure. While the period of 2010 to 2019 has seen the incidence rate of cancer at sites associated with tobacco and alcohol (e.g., lip and gums) decline, this has been counteracted with a rising incidence of cancer at sites associated with human papillomavirus (HPV) infection (e.g., oropharynx) [1].

Screening for HNC has traditionally occurred in physician and dentist offices through visual inspection and palpation for suspicious neck masses. Once diagnosed, treatment for HNC consists of a combination of surgery, radiotherapy, and chemotherapy. The exact regimen is driven by tumor site and stage, with multi-disciplinary tumor boards enhancing collaborative treatment approaches. Despite these efforts, the percent of locoregional failure is 15–50% [2,3,4]. Furthermore, the majority of HNC presents in advanced stages (stage III/IV), with associated worse survival in higher stages [5]. Thus, there is a need for improved diagnostic tools for earlier cancer detection.

Liquid biopsies isolate and analyze biological components released by tumors. These analytes are derived from various body effluents, including blood, urine, saliva, pleural fluid, and CSF, providing tumor genetic profiling rapidly and non-invasively. In HNC, liquid biopsies are commonly assessed from peripheral blood and oral saliva. Liquid biopsies can target tumor material ranging from extracellular vesicles to circulating tumor cells, with recent efforts focused on circulating tumor DNA (ctDNA), fragments of DNA released by tumor cells through physiologic and pathologic means [6]. CtDNA detection has garnered considerable interest in potential applications for cancer screening, management, and surveillance. In this review, we discuss the current state of ctDNA research for use as a HNC biomarker for screening, diagnosis, treatment response, and prognosis, as well as outline future directions and limitations of this field.

## 2. Methodologies for ctDNA Detection

### 2.1. ctDNA Characterization

DNA fragments were first observed in human plasma in 1948 by French scientists Mandel and Métais and described as cell-free nucleic acid [7]. Since then, several studies have attempted to elucidate the mechanisms behind how DNA fragments are released by healthy and tumor cells. It appears this may be carried out through both passive and active processes. The passive process is described as DNA expulsion, resulting from cell death through apoptosis or, as in the case of rapidly proliferating tumor cells, necrosis [8]. Conversely, the active process is not fully understood, but some contend that tumor cells release micro-vesicles that contain DNA fragments [9]. Circulating DNA, although detectable in healthy individuals, is considerably more concentrated in cancer patients [10]. Circulating extracellular DNA that is tumor-derived, referred to as ctDNA, often adheres to the surfaces of leukocytes and erythrocytes [11]. ctDNA that remains unbound is referred to as tumor-derived cell-free DNA (cfDNA).

### 2.2. ctDNA Detection Techniques

Detection techniques of ctDNA have evolved considerably since the time of Mandel and Métais in 1948 [7]. Currently, two approaches dominate the detection of ctDNA-targeted and untargeted approaches. Targeted approaches search for tumor-specific genetic sequences that are known in advance, such as driver mutations and integrated viral genes known to induce carcinogenesis (e.g., HPV and Epstein–Barr virus (EBV) genes). This approach includes polymerase chain reaction (PCR)-based techniques such as droplet digital PCR (ddPCR), and BEAMing (beads, emulsion, amplification, and magnetic). ddPCR relies on a water-oil emulsion droplet system that bypasses the limitations of serial dilutions required in other PCR technologies. This contributes to its high sensitivity in samples with low DNA levels with one study demonstrating DNA detection at concentrations as little as 37 copies per 20 µL [12,13,14,15,16,17]. BEAMing is a high-throughput droplet-based PCR that uses magnetized beads to separate DNA molecules which are further analyzed by flow cytometry [18]. BEAMing is notable for its ability to detect very rare genetic events such as mutant and wild type sequences present at ratios greater than 1:10,000 [19]. Both technologies are among the most commonly used assays for ctDNA detection and have been shown to have good agreement in ctDNA detection with limited discordancy [20]. Conversely, untargeted approaches do not require any prior knowledge of genetic alterations but are, consequently, less cost-effective [16]. This approach includes next generation sequencing (NGS)-based technologies including whole genome sequencing (WGS) and whole exome sequencing (WES). While WGS and WES can detect many more types of variants at once, they both also require a larger DNA input in the range of 200–1000 ng compared to 1 ng for BEAMing and ddPCR [21]. WGS and WES produce results as ratios, while BEAMing and ddPCR provide absolute quantification. Additionally, as will be discussed later, ctDNA epigenetic alterations are an active area of interest with gene methylation commonly investigated. ctDNA methylation is often quantified using methylation-specific PCR. This technique relies on purifying and sulphonating DNA and then performing a PCR reaction utilizing two pairs of primers, one specific for methylated DNA and the other for unmethylated DNA [22]. This is also a targeted approach as specific genetic sequences that are to be investigated must be known in advance.

Aside from differences in cost and technology, detection techniques may have varying performances in different body fluids. This was examined by Mattox et al., where plasma samples and oral rinses from 66 patients with HPV-positive oropharyngeal squamous cell cancer (OPSCC) were collected and analyzed for HPV ctDNA [23]. In plasma samples, NGS and ddPCR had similar sensitivities (68.3% and 69.8%, respectively), and both outperformed qPCR, which had a sensitivity of 20.6%. In oral rinses, NGS demonstrated significantly greater sensitivity at 75% compared to 8.3% and 2.1% for ddPCR and qPCR, respectively, indicating varying fluids may necessitate differing techniques to optimize detection. Notably, a key limitation in this study is that it examines isolated DNA previously extracted from banked plasma samples and oral rinses. DNA may have degraded over time, which may account for why this study found poorer performance by NGS, ddPCR, and qPCR than in other studies [24,25,26].

### 2.3. ctDNA Fluid Sources

ctDNA is a versatile biomarker and can be sourced from many fluid types, including pleural fluid for pulmonary adenocarcinomas and urine in urologic malignancies [27,28]. Studies examining ctDNA in HNC have largely focused on blood (plasma and serum) and saliva samples. Understandably, the anatomic location of the tumor can play a role in directing researchers on which bodily fluid is optimal for detecting ctDNA. This aspect was examined by Wang et al. in a study that enrolled 93 patients with head and neck squamous cell carcinoma (HNSCC) [24]. Saliva and plasma samples were collected from patients prior to a definitive treatment for primary HNSCC or salvage treatment for recurrent HNSCC. Digital PCR was used to detect tumor DNA with HPV sequences and Safe-SeqS (Safe-Sequencing System) for detecting low-frequency somatic mutations. For oral cavity SCC, saliva samples detected tumor DNA in 100% of patients versus only 80% of plasma samples. Conversely, plasma samples performed better in OPSCC patients by detecting tumor DNA in 91% compared to 47% of saliva. Similarly, plasma samples performed better than saliva samples in laryngeal (86% vs. 70%) and hypopharyngeal (100% to 67%) SCCs.

Sampling multiple fluid types in combination may further enhance detection. In a subset of patients (n = 43), Wang et al. demonstrated increased sensitivity for each tumor site when assays of plasma and saliva samples were combined [24]. Additionally, Ahn et al. also illustrated increased screening performance by combining analysis of ctDNA samples from multiple fluid types [29]. Here, 93 patients with OPSCC were enrolled and plasma samples and saliva rinses were collected. Quantitative PCR (qPCR) was conducted on samples to detect HPV E6 and E7 genes. Pretreatment saliva and plasma sources were found to have sensitivities of 52.8% and 67.3%, respectively, for detecting HPV-positive OPSCC. However, when both detection sources were combined, the sensitivity increased to 76.1%.

## 3. ctDNA Utility in HPV-Positive Head and Neck Cancer

### 3.1. HPV Characterization

The Centers of Disease Control and Prevention estimates that HPV now accounts for 70% of OPSCC in the United States [30]. HPV is spread mainly through sexual contact and exerts its carcinogenic effect by its oncogenes E6 and E7, which inactivate host tumor suppressor proteins p53 and pRb, respectively [31]. The importance of HPV status in OPSCC is established by studies demonstrating higher response rates to treatment and longer survival in patients with HPV-positive OPSCC, compared to patients with HPV-negative tumors [32,33]. Currently, the diagnosis of HPV-positive OPSCC is made by examining cytology specimens for either the presence of HPV DNA through PCR or in situ hybridization, or through detecting HPV surrogate markers such as host p16 overexpression, demonstrated through immunohistochemistry [34].

### 3.2. HPV ctDNA as a Biomarker for Screening and Diagnosis

ctDNA detection methods may offer an alternative method for diagnosing HPV-positive HNSCC (Table 1). In a prospective observational study, Siravegna et al. demonstrated that detection of HPV ctDNA may offer a noninvasive and cost-effective diagnostic approach for HPV-positive HNSCC with improved accuracy and reduced time to diagnosis [35]. A total of 61 patients with new or suspected diagnosis of untreated HNSCC were enrolled, as well as 70 HPV-negative controls. All patients with HNSCC underwent a standard clinical workup, which included fine needle aspiration and/or tissue biopsy of the primary tumor. The diagnostic success rate of the first diagnostic attempt was 72% with 28% of patients requiring a second diagnostic attempt with tumor biopsy to determine diagnosis. Conversely, serum HPV ctDNA detection for diagnosing HPV-positive HNSCC had a sensitivity of 98.4%, specificity of 98.6%, positive predictive value (PPV) of 98.4%, and negative predictive value (NPV) of 98.6%. When the composite performance of the standard clinical workup on first diagnostic attempt was compared to HPV ctDNA on the first diagnostic attempt, HPV ctDNA demonstrated improved diagnostic accuracy. Next, the authors conducted cost modeling comparing standard of care pathways with scenarios where HPV ctDNA was the diagnostic of choice. They estimated savings of 36–38% (USD 6227–USD 6667) per patient with HPV ctDNA diagnostics. Additionally, with existing molecular diagnostic turnaround times of 5 days, the authors estimated an HPV ctDNA diagnostic approach to shorten time to diagnosis by 63% (26 days). HPV ctDNA was additionally found to possess high sensitivity, even in a cohort with low disease burden (75% of patients with Stage I OPSCC), furthering interest as a screening tool.

Several barriers for screening for HPV-positive OPSCC have been identified, including its relatively low overall incidence, rendering even ideal biomarkers with low PPV [43]. Additionally, in the way cervical cancer possesses an identifiable precursor lesion for screening, nothing similarly has been described for OPSCC. However, in a retrospective case–control study, Rettig et al. have shown that HPV ctDNA detection can occur several years prior to the diagnosis of HPV-positive OPSCC, suggesting HPV ctDNA positivity could serve as a surrogate precursor lesion [36]. Of the 10 patients with HPV-positive OPSCC enrolled, 3 had early detectable HPV ctDNA in plasma collected at a median time of 30.5 months prior to diagnosis. Neither the cases with HPV-negative OPSCC nor any of the 100 healthy controls had detectable HPV ctDNA in their plasma. While the generalizability of these findings is limited by the low number of cases, these findings demonstrate for the first time that HPV ctDNA can be detected in plasma years before a clinical diagnosis of HPV-positive OPSCC. The authors also demonstrated that HPV ctDNA can have high specificity with zero false positives reported. A cross-sectional analysis also found similar specificity for plasma-derived HPV ctDNA [37]. The authors enrolled 408 healthy participants without HNC but at heightened risk for HPV-related cancer, as determined by lifestyle factors. PCR conducted on plasma samples from participants did not detect any oncogenic HPV ctDNA.

### 3.3. HPV ctDNA as a Biomarker for Surveillance

Studies have begun examining the ability of HPV ctDNA plasma presence to detect disease recurrence in HPV-positive OPSCC with promising accuracy [44,45]. In a prospective clinical trial of 115 HPV-positive OPSCC patients, Chera et al. demonstrated that two consecutive positive HPV ctDNA blood tests during posttreatment surveillance was highly indicative of disease recurrence [26]. After a median follow-up time of 23 months, 15 patients developed biopsy-proven recurrence, all of whom had two consecutively positive HPV ctDNA tests during surveillance, with a sensitivity and specificity 100% and 99%, respectively. Another promising result was that the median lead time from the first positive HPV ctDNA to biopsy-proven recurrence was 3.9 months.

PET-CT imaging has remained a controversial surveillance modality, as it has yet to show survival advantage, and in some studies has been shown to have low PPV for detecting locoregional failure in HPV-positive OPSCC [46,47]. Tanaka et al. demonstrated that concomitant HPV ctDNA blood tests with PET-CT imaging, however, could improve recurrent/residual disease detection [38]. A total of 35 patients with HPV-positive OPSCC were enrolled in this prospective cohort study after completing chemoradiotherapy. After a median follow-up of 21 months, 9 patients had treatment failures. PET-CT imaging that displayed incomplete metabolic response had a 4.7-fold increase in risk of residual disease compared to patients who had complete metabolic response. However, with combined imaging and liquid biopsy results, positive HPV ctDNA levels and incomplete metabolic response on PET-CT portended a 138.8-fold increased risk of residual disease when compared to patients with non-detectable HPV ctDNA levels and incomplete metabolic response on PET-CT. Another study with a small cohort found similar improvement in the detection ability of post-chemoradiotherapy residual disease with combined PET-CT imaging and HPV ctDNA detection [48].

Other studies have begun determining the absolute quantification of HPV ctDNA levels in plasma specimens and analyzing its kinetic clearance pattern to predict recurrent/residual disease. Chera et al. recruited 103 patients with HPV-positive OPSCC who had undergone chemoradiotherapy in a multi-institutional prospective biomarker trial [39]. The authors found that patients with a baseline HPV ctDNA plasma level of >200 copies/mL and who had greater than 95% of HPV ctDNA clearance by week 4 post-treatment had a greater likelihood of disease control. Elsewhere, Haring et al. suggest that the percent change in HPV ctDNA levels during chemotherapy correlates with the radiographically determined treatment response [40]. The authors demonstrated that HPV ctDNA levels showing an increase greater than 60% between baseline and cycle 3 of chemotherapy were predictive of progressive disease with a sensitivity and specificity of 89%. Post-operative HPV ctDNA levels have also been shown to predict residual disease risk. O’Boyle et al. showed that post-operative day 1 HPV ctDNA plasma levels of 1 copy/mL correlated with the lowest risk of residual disease, while 100 copies/mL correlated with higher incidence of pathologic risk factors such as extranodal extension and number of lymph nodes involved; these findings are also supported in another study by Routman et al. [41,42]. While future studies are needed to validate these findings, they do provide encouraging glimpses into how HPV ctDNA can potentially serve as a biomarker for guiding personalized treatment decisions, such as the need for adjuvant therapy or treatment deintensification.

## 4. ctDNA Utility in EBV-Associated Nasopharyngeal Carcinoma

### 4.1. EBV Characterization

The Epstein–Barr virus has been associated with several different malignancies, including nasopharyngeal carcinoma (NPC) [49,50]. It has been determined to affect around 85–95% of the healthy population and has been endemically linked with NPC in Southeast Asia [44]. Unfortunately, NPC is frequently diagnosed at later stages due to the inaccessible nature of the post-nasal space and often atypical presentation, leading to poorer patient outcomes [45].

### 4.2. EBV ctDNA as a Biomarker for Screening

The role for plasma EBV ctDNA load in the detection and screening utility of NPC has been well-characterized in the endemic literature [51,52]. It continues to be a role vigorously investigated (Table 2). The landmark prospective investigation conducted by Lo et al. found elevated EBV ctDNA loads in 55/57 (96.0%) patients with NPC compared to 3/43 (7.0%) of controls, establishing the value of plasma EBV ctDNA as a biomarker for screening NPC [53]. Similar results were reproduced in a non-endemic population but with a lower reported sensitivity (75.0%) [54]. Since then, several other EBV-associated biomarkers have been studied, including EBV viral capsid antigen and EBV early antigen IgA serology. Although the effectiveness of these other biomarkers has been inconsistently reported in the literature, they may prove to be beneficial in the detection of earlier stages of NPC [55,56,57]. These alternative biomarkers are important to consider, since EBV ctDNA load may not be as sensitive in detecting earlier compared to later-stage NPC [58]. On the other hand, several large prospective investigations in endemic areas have reported that the overall sensitivity and specificity of EBV ctDNA load in screening for NPC to be quite promising: at 86.8–97.1% and 90.0–98.6%, respectively [58,59]. Miller et al. reported through a hypothetical cohort that the combined usage of EBV ctDNA load and EBV serology would be a cost-effective option that could improve the 10-year overall survival from 71.0% to 86.3%, suggesting a potential advantage in combining these screening modalities [60]. However, more consistent methodological means are still needed to reduce inter-laboratory procedural variabilities, including DNA extraction protocols, and set EBV ctDNA load screening cutoff values [61,62].

### 4.3. EBV ctDNA as a Biomarker for Surveillance and Prognosis

In addition to being positively correlated to clinical staging, EBV ctDNA load has also been extensively studied as an independent factor in monitoring treatment response [63,69,70,71]. To et al. determined that the median half-life of EBV DNA during surgical resection was 139 min, proving that EBV ctDNA is derived from the NPC tumor body [72]. Furthermore, the clearance of EBV ctDNA after chemoradiotherapy has been well documented in the literature and has been used as an indicator for treatment response [64,65,70]. Resurgences in EBV ctDNA levels after curative therapy have been associated with disease recurrence or residual malignancy, which suggests a possible use for EBV ctDNA measurements in disease surveillance [51,69,73]. Even when measured before treatment initiation, EBV ctDNA levels were significantly associated with metastatic disease [66,74]. In a meta-analysis of 16 pooled studies, Liu et al. found that the pooled hazard ratio for both locoregional recurrence and distant metastases in patients with higher pre-treatment EBV ctDNA levels were 3.12 and 3.68, respectively [69]. It should be mentioned that there were several different cutoffs for higher pre-treatment EBV ctDNA for the studies in this meta-analysis, ranging from 307 to 20,000 copies/mL. As with screening, further standardization of EBV ctDNA cutoff values and inter-laboratory methodological means has yet to be established.

EBV ctDNA levels are negatively correlated with survival outcome measures. Detectable pre-treatment plasma EBV ctDNA was found to be associated with poorer survival outcomes when compared to undetectable cases [63,67,70,75]. This trend has also been demonstrated in non-endemic investigations [62,76]. Additionally, several investigations found that even among cases with detectable EBV ctDNA, higher levels of plasma EBV ctDNA were associated with poorer survival outcomes. It should be noted that there was a high amount of variability regarding the cutoffs for higher levels of EBV DNA, with a range between 1500 and 7000 copies/mL [64,65,67,70,71]. In Liu et al.’s meta-analysis, higher EBV ctDNA loads were associated with poorer overall survival (OS) and disease-free survival (DFS) with hazard ratios of 3.0 and 2.4, respectively [69]. Several studies have also found mid-treatment and post-treatment detectable EBV ctDNA levels to be associated with poorer survival outcomes [64,65,70,77]. While there was a large overlap between patients with detectable mid and post-treatment EBV ctDNA levels, patients with increased mid-treatment but undetectable post-treatment EBV ctDNA levels were still found to have worse outcomes than individuals with undetectable mid-treatment levels [77]. Additionally, Chan et al. found patients undergoing radiotherapy with EBV ctDNA half-lives of ≥15 days to have significantly poorer OS, progression-free survival (PFS), and distant metastasis-free survival (DMFS) than those with more expedient clearance, suggesting a temporal relationship between viral DNA clearance and survival outcomes [78]. Although radiotherapy was found to decrease EBV ctDNA levels and improve survival outcomes in patients with NPC, the data concerning chemotherapy are mixed [68,79]. These data show that EBV ctDNA may play a significant role in determining patient prognosis, for both survival outcomes and disease relapse or metastases.

## 5. Non-Viral ctDNA Biomarkers

### 5.1. ctDNA Somatic Mutations

While still in its preliminary stages of implementation, several studies have established the potential of using ctDNA in detecting somatic mutations to screen for HNSCC. The mutational landscape of HNSCC has been well-investigated recently in a number of large sequencing cohorts, including The Cancer Genome Atlas [80]. Recurring somatic gene mutations in HNSCC have been identified, including those in high frequencies (e.g., *TP53* mutations occur in the majority of non-HPV HNSCC), making these potential targets for ctDNA approaches. Other frequently mutated genes and pathways include *CDKN2A*, *PIK3CA*, *CASP8*, and *NOTCH1* [81,82,83,84,85,86,87,88,89,90]. As expected, the most common mutated gene found through ctDNA across most studies was *TP53*, which was found to be present in 18–31% of patients [91,92,93], barring one study where plasma samples were stored for 4 years at 8% [92]. Notably, this rate is significantly lower than the mutation rate of *TP53* in HNSCC (>70%), suggesting some current limitations in using ctDNA for *TP53* mutations as a biomarker target. Additionally, in an investigation of 36 cases, the concordance between saliva and tumor sample analysis was found to be only 11% for TP53 mutation detection [93]. In a later study involving 39 samples, the sensitivity of ctDNA was once again found to be quite limited, with a concordance of 19% between liquid and solid biopsies [94]. Importantly, the majority of the literature presented aggregate data of total somatic mutations, without further information on mutational distributions [24,95,96]. Furthermore, the few studies that do provide these categorical data are limited by small sample sizes. In Hudeckova et al.’s systematic review, *PIK3CA*, *CDK2NA*, *NOTCH1*, and *CASP8* mutations in ctDNA were reported to be present in 31%, 6%, 3%, and 3% of cases, respectively [92]. However, these data are based on single-institution studies and may not be generalizable to other populations [93,97].

The sensitivity of ctDNA in detecting somatic mutations may be improved by utilizing both plasma and saliva samples. Wang et al. were able to identify all 20 patients with early stage (I–II) disease with this combined saliva and plasma methodology, demonstrating the promise of using this methodology in HNSCC screening [24]. However, the sensitivity of ctDNA in detecting somatic mutations in HNSCC is limited in comparison to that of other solid cancers. Mes et al. reported that of the 27 patients found to have somatic mutations on solid biopsy sequencing, only 18 were found to have at least 1 corresponding mutation with their plasma counterparts [95]. Additionally, there have been no published randomized controlled trials (RCT) that have evaluated the sensitivity and specificity of ctDNA in the detection of somatic mutations to date. Therein, significant improvements must be made prior to using somatic ctDNA mutations as a screening tool in HNSCC detection.

While the effectiveness of somatic ctDNA mutations in prognostication has been confirmed across several different cancer types [98,99,100], there have been conflicting or limited data pertaining to colorectal, pancreatic, and breast cancer [101,102,103]. Similarly, the literature on using this biomarker as a prognosticator has also been inconsistent regarding HNSCC. In a 2017 investigation by Perdomo et al., there were no differences in survival outcomes between patients with and without detectable somatic ctDNA mutations [93]. In contrast, Porter et al.’s investigation found increased frequencies of somatic ctDNA mutations in those with metastatic disease when compared to those with recurrent disease, suggesting a positive relationship between these biomarkers and disease progression [104]. Kogo et al. analyzed the temporal relationship between ctDNA across multiple time points, including immediately postoperatively and every 2–3 months afterwards [96]. Here, the presence of mutated ctDNA genes immediately postoperatively were associated with significantly worse OS. Furthermore, in patients with initially negative postoperative ctDNA who subsequently developed positive results also resulted in significantly decreased OS. Despite this, RCTs and more rigorous prospective studies will need to be performed prior to incorporating this practice into routine clinical practice.

### 5.2. ctDNA Gene Methylation

An increasing body of work has highlighted a correlation between primary tumor gene methylation and ctDNA gene methylation in HNSCC. In a systematic review by Pall et al., ctDNA methylations were significantly increased in HNSCC patients compared to controls [105]. The authors further found that increasing the number of ctDNA genetic methylations resulted in an increase in diagnostic sensitivity and accuracy. Among these, the most frequently studied gene methylations were *TP53*, *CDKN2A*, *DAPK1*, *RASSF1*, and *p15*. Wong et al. similarly used a methylation-specific PCR to evaluate the methylation status of *p16* and *p15* genes in 73 HNSCC surgical specimens and found that methylated *p16* and *p15* DNA levels were significantly higher in the plasma of HNSCC patients compared to normal controls [106]. Interestingly, significantly higher levels of *p15* methylation were found among histologically normal tissues of HNC patients with chronic tobacco and alcohol use habits compared with non-smokers and non-drinkers in their population, thus suggesting that differential plasma methylation levels may be potentially useful biomarkers in screening high-risk patients for early-stage disease and monitoring treatment response. In a separate study which compared the serum from patients with HNSCC and healthy controls, hypermethylation of *EDNRB* was found to be a highly specific but not sensitive serum biomarker for HNSCC [107]. Ovchinnikov et al. demonstrated that methylation in the promoters of *RASSF1A*, *DAPK1*, and *p16* genes was able to detect tumor presence in the DNA isolated from the saliva of HNSCC patients with an overall accuracy of 81% compared to healthy, non-smoker controls [108]. Righini and colleagues showed similar associations between hypermethylated gene targets in head and neck tumor and paired saliva samples [109].

While many assays designed to evaluate hypermethylated gene targets are in use, the diagnostic accuracy, sensitivity, and specificity between studies vary widely. In addition, there is no consensus regarding either the ideal number or specific biomarker targets among methylation series. Thus, further investigations and clinical trials are required to identify the hypermethylated ctDNA gene mutations affected among HNC patients and to validate their incorporation into future screening tools.

Similar epigenetic relationships between EBV DNA and NPC have also been documented, with ctDNA hypermethylation status sharing similarities with the primary tumor. In an analysis of serum ctDNA collected from 40 NPC patients and 41 age- and sex-matched healthy subjects, gene promoter hypermethylation of five key tumor suppressor genes was appreciated in as many as 64.9% of cases. This demonstrates that screening DNA hypermethylation of tumor suppressor genes represents a possible avenue for the early detection of NPC [110]. In a unique study utilizing methylation-sensitive high resolution melting (MS-HRM) assays in nasopharyngeal biopsies, brushings, and cell-free plasma from NPC patients, Yang et al. found that the DNA methylation panel was characterized by a higher sensitivity and specificity when compared to plasma EBV DNA among early stage I and II NPC patients [111]. Furthermore, using the selected MS-HRM assay for plasma and nasopharyngeal brushing, DNA methylation significantly increased the detection rate of NPC among all disease stages, as well as local recurrence. Thus, the combination of a plasma DNA methylation panel and tests of EBV DNA may increase the screening potential and detection of NPC in both early and late-stage patients.

## 6. Future Directions and Challenges

Within the field of head and neck oncology, there has been a recent emphasis on refining techniques associated with liquid biopsies and non-invasive identification of both viral and non-viral ctDNA, epigenetic modifications, and numerous other biomarkers designed to detect cancer and characterize disease status.

### 6.1. Other Liquid Biopsy Targets

Indeed, there has been an increasing focus on expanding the targets of liquid biopsies, which suggests a promising future direction for this field. Exosomes (EXOs) have recently been identified as a potential biomarker for the identification of HNSCC and for characterizing disease status. EXOs, or virus-sized extracellular vesicles ranging from 30 to 150 nm in diameter containing proteins, miRNA, mRNA, and DNA, are released by cells and remain detectable in saliva, blood, urine, and cerebrospinal fluid [112,113]. As tumor cells are highly active in producing EXOs, they have recently been demonstrated to accumulate within the tumor microenvironment, with notable differences between HPV-positive and HPV-negative cell lines [114]. More specifically, patients with early or advanced HNSCC may show significantly higher plasma EXOs when compared to healthy controls [115,116]. Monitoring of EXOs has also been shown to have further implications on the tumor microenvironment. The programmed death-ligand 1 (PD-L1) and programmed death-1 (PD-1) pathway is central to this understanding, with many tumor cells able to evade immune surveillance via the upregulation of PD-L1 within HNSCC [117,118]. Theodoraki and colleagues isolated exosomes from the plasma of 40 HNSCC patients and tested for soluble PD-L1 through ELISA in addition to flow cytometry, and additional PD-1/PD-L1 staining [119]. The authors found that levels of PD-L1 carried by exosomes correlated with disease stage, activity, and lymph node status. Notably, blocking of PD-L1+ exosome signaling to PD-1+ T-cells attenuated immune suppression. This research provides further evidence that circulating PD-L1 expression on EXOs may be harnessed as a future non-invasive biomarker and used to monitor for the presence of disease, tumor stage, and progression [120].

Various other metabolites and biomarkers have been shown to concentrate within EXOs and show additional promise in the identification and monitoring of HNC. Within the blood and saliva of HNSCC patients, microRNAs (miRNA)—or small, non-coding mRNA molecules—have been noted to mediate various cellular processes in tumorigenesis [121]. Various investigators have demonstrated the role of miRNA and salivary-derived EXOs as novel HNSCC biomarkers [122,123,124,125]. He et al., using miRNA microarray analysis to compare salivary exosomes between patients with and without oral cavity SCC (OCSCC), identified a total of 109 miRNAs to be more than 2-fold altered; the authors specifically found salivary exosomal miR-24-3p to increase the proliferation of malignant tumor cells and accurately identified patients with OCSCC [122]. More generally, miRNA has also been shown to correlate with disease status, with miR-31 levels significantly elevated among patients with OCSCCC with a marked reduction following tumor extirpation [126]. These studies emphasize the importance of EXOs, miRNA, and similar metabolites as potential biomarkers in HNSCC.

Other intriguing analytes under active investigation are circulating tumor cells (CTCs) (Table 3). These are cancer cells that are derived from a primary tumor or metastatic sites that have entered the vasculature. Studies have shown that serial measurements of CTCs in blood samples could prove useful in monitoring disease status during and after treatment [127], while other studies have illustrated it may have predictive value for regional metastasis in HNC [128]. However, conflicting performances in predicting DFS and OFS in HNSCC suggest that it is still an imperfect prognostic biomarker [129,130]. A challenging limitation of CTCs as a liquid biopsy biomarker is that it is difficult to detect and isolate from whole blood samples, as CTCs are very rare with approximately 1–100 CTC/mL among the billions of red blood cells [131]. However, new platforms are emerging that attempt to overcome this limitation by using a label-free inertial microfluidic approach that reports increased detection of CTCs (3–133 CTC/mL of blood) than older platforms [132].

### 6.2. Assessing Limitations and Optimizing ctDNA

Preliminary work in the liquid biopsy area has shown promise in translating this science into clinically useful measures and possible screening tools. Despite these advances, significant challenges remain. ctDNA and other liquid biomarker techniques will benefit from improved sensitivity, specificity, and overall accuracy required for widespread, meaningful clinical use. While solid tissue biopsies presently remain the gold standard and reference for most ctDNA analyses, as demonstrated by the above studies, even this presents controversy. ctDNA may be derived from areas of tumors that were not biopsied from the index lesion and may contain a divergent genomic composition limiting targeted approaches [133]. Questions remain how representative liquid biopsies can be of solid tumors with respect to intra-tumor heterogeneity. This may not present an issue in virally mediated HNC as HPV and EBV ctDNA are specific biomarkers that can reliably be detected. However, this does pose a challenge in non-viral HNC, as driver mutations may be heterogeneously distributed throughout a primary tumor. Liebs et al. illustrated the concordance of the mutational profiles between tumor tissue and cfDNA in HNC patients to only be 11%, highlighting the significant heterogeneity of HNC [134]. While CTCs did not perform better, they were interestingly able to detect specific mutations not detected in the tissue samples, suggesting a potential advantage of liquid biopsies. While they may not fully capture the intra-tumor heterogeneity and tumor microenvironments of primary tumors, liquid biopsies may have the potential to detect genomic data from micrometastases and other subclones that contribute to treatment resistance [135]. For example, in patients with metastatic breast cancer, Chu et al. utilized ctDNA sequencing to detect ESR1 mutations, which would suggest tumor resistance to endocrine therapy, which was not identified in the corresponding solid biopsies of metastatic lesions [136]. This highlights a similar limitation of spot core biopsies and fine-needle aspirations of solid tumors, as they also do not capture tumor heterogeneity or specific niches of tumor microenvironment. Nonetheless, the pursuit of capturing tumor heterogeneity is an important field of research, and tumor heterogeneity likely contributes to why reliable assessable mutations in non-virally associated HNC have not yet been identified. Thus, further studies are needed to clarify these associations and identify mutations, cell surface markers, or other metabolites which may increase the ability to more accurately isolate liquid biopsy targets among various HNC tumors and subsites. Potential opportunities include ctDNA mutational panels, or expression signatures.

Currently, numerous assays to detect ctDNA exist; however, no single ideal technique or assay has been universally adopted. Concerns for inter-laboratory variability in accuracy and interpretation have hobbled mainstream clinical adoption. Further studies evaluating agreement of different assays in ctDNA detection are needed, similar to how O’Leary et al. demonstrated good agreement between BEAMing and ddPCR [20]. This will clarify whether there is sufficient reproducibility for clinical use and if analyses among different studies can appropriately be made. Additionally, by improving the associated sensitivity and specificity of these techniques and further understanding ctDNA biology, more accurate detection of HNC, disease burden, surveillance, and prognostication may be possible [137].

Through combinatorial approaches and future technological refinements, integration of ctDNA data with various other circulating biomarkers may ultimately broaden the scope of ctDNA applications in HNC and improve its utility as a clinical tool. Several studies have investigated the combined role of ctDNA and CTC detection in solid tumors, but to date, none have been conducted in a solely HNC sample [132,138]. Furthermore, advancements in platforms similar to Bu et al. that can simultaneously capture ctDNA, exosomes, and CTCs and provide combinatorial analysis of their expression profiles through machine learning will ease this path of investigation [139].

ctDNA has been increasingly studied as a biomarker for HNSCC post-treatment surveillance and used to assess residual disease, monitor for recurrence, and stratify patients at increased risk for relapse of disease [26,140,141,142]. Despite the early integration of ctDNA and similar biomarkers in treatment algorithms, further large-scale prospective and randomized clinical trials in HNC cohorts are needed to fully integrate ctDNA data into treatment stratification paradigms. Indeed, several clinical trials are underway including a phase II trial using ctDNA testing to determine the optimal time to begin routine treatment in patients with HPV-positive HNC [143,144,145]. While clinical validation of ctDNA use in non-viral HNC is continuing [146], clinical validation in virally mediated HNC cohorts have demonstrated excellent detection of viral ctDNA as biomarkers for screening, treatment response and surveillance (as noted above by Chera et al. in HPV-related HNC and Lo et al. in EBV-related HNC, among others; Table 1 and Table 2) [26,53]. Additionally, although not yet standard of care, HPV ctDNA is already being used in patient care as a biomarker [147]. ctDNA assays have also already been clinically validated in several cancer types, including non-small-cell lung cancer, colorectal cancer, and pancreatic cancer, representing the robustness of the technology and ctDNA as a biomarker [148,149,150]. The studies presented in this review do reveal areas in need of improvement in the field of ctDNA usage for HNC, including more cost-effective detection options, enhanced accuracy, expanded gene targets, and broadened combinatorial approaches with other liquid biopsy targets. Yet, with the break-neck speed of research in this field, these areas are to be strengthened quickly. While the American Society of Clinical Oncology (ASCO) and College of American Pathologists published a joint review in 2018, concluding at the time that ctDNA assays did not possess the evidence to suggest clinical utility outside of clinical trials [151], a re-evaluation of new literature is warranted and may soon suggest otherwise.

## 7. Conclusions

In an era of rapid genetic sequencing through NGS and advancements in PCR-based techniques, personalized care of head and neck cancer is within reach for optimizing disease management. ctDNA testing holds encouraging capabilities for facilitating screening, diagnosis, treatment stratification, and surveillance in a non-invasive and repeatable manner. Viral and non-viral ctDNA biomarkers, as well as other liquid biomarkers, may prove integral in early detection and improved treatment outcomes. While further investigation with larger prospective studies and randomized clinical trials is warranted before assimilating ctDNA testing into management paradigms, continuing advancements in this field render further studies of ctDNA as highly anticipated.

## Figures and Tables

**Table 1:** Key studies examining HPV ctDNA as a biomarker for HPV-positive HNSCC.

Reference	Study Design	Sample Size	Findings/Strengths	Limitations
[26]	Prospective	115	- HPV ctDNA plasma detection has high NPV and PPV for disease recurrence surveillance - Phase II clinical trial - Multi-institutional study	- 29 patients did not have pre-treatment blood samples available
[35]	Prospective	140	- Demonstrates diagnostic capacity of HPV ctDNA testing as cost-effective with shorter diagnostic interval - Prospectively conducted	- Small sample size - Observational in design - Single institutional study
[36]	Retrospective	112	- First report to demonstrate HPV ctDNA detection years prior to cancer diagnosis - HPV ctDNA detection demonstrated high specificity for diagnosis of HPV-positive cancer	- Small sample size of only 12 cases - Retrospective design - Single-institutional study
[37]	Cross-sectional analysis	408	- HPV ctDNA testing in plasma had 100% specificity in healthy people - Large sample size	- Limited to single timepoint - Single institutional study
[38]	Prospective	35	- HPV ctDNA testing increases accuracy of post-treatment surveillance when combined with PET-CT imaging	- Small sample size - Single institutional study
[39]	Prospective	103	- Identified a favorable and unfavorable clearance profile that can predict CRT treatment response - Demonstrated utility of HPV ctDNA load to select patients for de-intensified therapy - Multi-institutional study	- Limited follow up
[40]	Prospective	16	- Serial HPV ctDNA loads can be used to measure treatment response with potential for guiding treatment intensification/deintensification	- Small sample size - HPV ctDNA only detected in 75% of patients with HPV-positive OPSCC
[41]	Prospective	33	- Clearance kinetics of HPV ctDNA can be used to identify patients at increased risk of recurrence and those who may benefit from adjuvant treatment.	- Small sample size - Single institutional study - Short follow-up
[42]	Prospective	159	- Post-op HPV ctDNA levels have prognostic value for RFS and OS	- Pre-op to post-op HPV ctDNA level comparisons to a small subset of patients - Post-op blood collections for HPV ctDNA analysis collected at varying timepoints affecting understanding of ctDNA kinetics and quantity

RFS = Recurrence-free survival; OS = Overall Survival.

**Table 2:** Key studies examining EBV ctDNA as a biomarker for EBV-associated NPC.

Reference	Study Design	Sample Size	Findings/Strengths	Limitations
[50]	Prospective	1363	- EBV ctDNA detectable group had a 10-fold higher incidence for NPC than undetectable group - Large sample size	- Did not retest or monitor EBV DNA fluctuation - Endemic population
[57]	Prospective	523	- EBV cfDNA load levels had poorer performance in screening for NPC than EBV IgA titers	- Endemic population - Only first-degree family members of NPC patients
[58]	Prospective	773	- Detectable EBV ctDNA levels had lower sensitivity for screening for early stage NPC than advanced stage - Large study population	- Endemic population - Not all high-risk patients underwent diagnostics
[59]	Prospective	20,174	- EBV ctDNA detection in plasma samples had a sensitivity and specificity of 97.1% and 98.6% in screening for NPC - Large sample size - 2 different measurements to confirm EBV ctDNA	- Endemic population - Male only - Short 2-year follow-up interval
[63]	Retrospective	480	- Undetectable EBV ctDNA levels before treatment was associated with earlier T and N classification NPC	- Retrospective design - Single-institutional study
[64]	Retrospective	278	- After induction chemotherapy, detectable EBV ctDNA levels were associated with worse 3-year OS, DMFS, and DFS than undetectable levels	- Endemic population - Single-institutional study
[65]	Retrospective	637	- Pre-treatment EBV ctDNA loads >1500 copies/mL and post-treatment detectable EBV ctDNA were both associated with higher risk for recurrence and mortality	- Endemic population - Retrospective - Limited follow-up
[66]	Retrospective	4469	- Patients with large EBV ctDNA load had higher tendency for distant metastases - Large sample size	- Only pre-treatment EBV DNA measured
[67]	Retrospective	1124	- EBV ctDNA load > 4000 copies/mL during chemotherapy and IMRT treatment was an independent risk factor for OS - Large sample size	- Single-institutional study - Patients with heart, liver, renal, and/or hematologic comorbidities were excluded
[68]	Prospective	260	- Undetectable EBC ctDNA at 8 weeks and 6 months post-IMRT was associated with longer 3-year survival endpoints	- Endemic Population

OS = Overall survival; DMFS = Distant metastasis-free survival; DFS = Disease-free survival; IMRT = Intensity-modulate radiation therapy.

**Table 3:** Key characteristics of liquid biopsy targets for HNC.

	ctDNA	EXO	CTC
**Fluid Source**	Blood; Saliva	Blood; Saliva	Blood
**Concentration in Fluid**	Moderate	High	Low
**Sensitivity**	Higher	Higher	Lower
**Specificity**	Higher	Lower	Variable
**Applications**	Screening; Prognosis; Treatment selection; Post-treatment surveillance	Prognosis; Post-treatment surveillance	Prognosis; Treatment selection
